# Vaccines and Vaccination against SARS-CoV-2: Considerations for the Older Population

**DOI:** 10.3390/vaccines9121435

**Published:** 2021-12-04

**Authors:** Birgit Weinberger

**Affiliations:** Institute for Biomedical Aging Research, Universität Innsbruck, 6020 Innsbruck, Austria; birgit.weinberger@uibk.ac.at

**Keywords:** SARS-CoV-2, COVID-19, vaccine, vaccination, elderly, aging

## Abstract

Age is among the most prominent risk factors for developing severe COVID-19 disease, and therefore older adults are a major target group for vaccination against SARS-CoV-2. This review focusses on age-associated aspects of COVID-19 vaccines and vaccination strategies, and summarizes data on immunogenicity, efficacy and effectiveness of the four COVID-19 vaccines, which are licensed in the US and/or Europe; namely, the two mRNA vaccines by BioNTech/Pfizer (BNT162b2) and Moderna (mRNA-1273), and the adenovector vaccines developed by AstraZeneca/University Oxford (ChAdOx1-nCoV-19, AZD1222) and Janssen/Johnson&Johnson (Ad26.COV2-S), respectively. After very high protection rates in the first months after vaccination even in the older population, effectiveness of the vaccines, particularly against asymptomatic infection and mild disease, declined at later time points and with the emergence of virus variants. Many high-income countries have recently started administration of additional doses to older adults and other high-risk groups, whereas other parts of the world are still struggling to acquire and distribute vaccines for primary vaccination. Other vaccines are available in other countries and clinical development for more vaccine candidates is ongoing, but a complete overview of COVID-19 vaccine development is beyond the scope of this article.

## 1. Introduction

The immune system undergoes characteristic changes with age, which lead to dysregulation and functional deficits of many immune mechanisms. Functional changes of innate immune cells impact the first line of immunological defense as well as the induction of adaptive immune responses. In the context of vaccination, innate immune cells at the site of injection are crucial for successful immune responses. Neutrophils contribute to a proinflammatory environment at the site of infection or vaccine delivery and play an important role in the recruitment and activation of other innate immune cells, e.g., monocytes/macrophages and dendritic cells (DC). Neutrophils from older persons have been shown to display reduced chemotaxis, altered signal transduction following antigen recognition and aberrant cytokine production [1]. Similar defects can be observed in monocytes/macrophages and DCs. In addition, their capacity to process and present antigen to T cells is decreased e.g., due to alterations in the upregulation of MHC-proteins and other stimulatory molecules after antigen contact [2].

Alterations in hematopoiesis and thymic involution lead to substantial changes in the composition of the T cell compartment with age. The output of newly generated naïve T cells decreases whereas antigen-experienced, highly-differentiated T cells, which have been repeatedly stimulated by antigen, accumulate. These cells show a reduced response to antigenic stimulation, produce preferentially proinflammatory cytokines, and are restricted in their diversity [3,4,5]. The composition of the B cell pool also changes with age, and an increase of B cells with lower affinity or autoreactivity can be observed. Lower numbers of antibody-producing plasma cells can be observed after vaccination and intrinsic defects of B cells, such as reduced isotype switch and affinity maturation, further contribute to lower antibody responses [6]. Interactions of B cells with follicular T helper cells in germinal centers are crucial for antibody responses. Age-related deficits of this cell type need to be taken into account, and have recently been summarized elsewhere [7].

An extensive review of immunosenescence and other factors influencing immune responses to vaccination is beyond the scope of this article, but can be found elsewhere [8,9,10,11,12]. As a consequence of immunosenescence, incidence, morbidity, and mortality of many infectious diseases are increased in older adults [13]. Additional risk factors for infections include chronic comorbidities, such as cardiovascular disease, kidney disease, diabetes or malignancies, which are frequent in the older population, and obesity, as well as immunosuppressive treatments, e.g., in the context of organ transplantation, chemotherapy or immunomodulatory treatment of autoimmune disease. These phenomena have been observed and studied (e.g., for influenza, pneumococcal disease, and herpes zoster) for a long time [14,15,16]. At the same time, immunogenicity and clinical efficacy/effectiveness are lower in older adults for many, but not all vaccines [17,18,19]. In the current SARS-CoV-2 pandemic very heterogeneous clinical presentations of COVID-19 disease have been observed, ranging from asymptomatic to severe and fatal disease. Identification of risk factors is crucial for mitigation strategies, and a plethora of studies addressed these questions early in the pandemic. They found that the risk for severe disease and death from COVID-19 is highest in older adults, persons with underlying co-morbidities, and obese individuals [20,21,22,23]. A systematic review summarizing 76 studies and a total of more than 153,000 patients confirmed these findings and reported age above 75 years (OR: 2.65, 95% CI: 1.81–3.90), male sex (OR: 2.05, 95% CI: 1.39–3.04) and severe obesity (OR: 2.57, 95% CI: 1.31–5.05) as the greatest risk factors for severe disease. When considering mortality as the outcome, the risk associated with age >75 is elevated further (OR: 5.57, 95% CI: 3.10–10.00) [20]. Another study compiled data from several countries and calculated that COVID-19 hospitalization rates increase exponentially with age and double every 16 years [24]. In addition to the acute course of infection, long-lasting symptoms and health sequelae have been reported after recovery from COVID-19, which are referred to as “long COVID” or “post COVID syndrome”. First reports highlighted the long-term sequelae mostly for younger adults, but now studies suggest that the risk for developing long COVID also increases with age [25,26]. 

Vaccine development was initiated rapidly after the identification of the novel coronavirus SARS-CoV-2 by many companies and organizations worldwide. It became evident early in the pandemic that efficient vaccines would be essential to control the virus and that older adults are an important target group for vaccination against SARS-CoV-2. Older adults were among the first to receive vaccines against SARS-CoV-2 when they became available as most national vaccination programs prioritized this age group. 

This summary focusses on SARS-CoV-2 vaccines currently licensed or authorized in Europe, namely the mRNA-based vaccines of BioNTech/Pfizer (Comirnaty; BNT162b2) and Moderna (Spikevax; mRNA-1273) and the adenoviral vectors of Oxford/AstraZeneca (chimpanzee adenovirus ChAdOx1; Vaxzevria; AZD1222) and Johnson&Johnson/Janssen (human Adenovirus-26; COVID-19 Vaccine Janssen; Ad26.COV2-S) and highlights their safety, immunogenicity, efficacy, and effectiveness in older adults. Except for AZD1222, they are also licensed or authorized in the US, and all four vaccines are also licensed and used in many other countries. Three additional vaccines are approved for use by the World Health Organization (WHO) and are used in many countries, namely, Covishield, which is the same formulation as the Oxford/AstraZeneca vaccine but produced by the Serum Institute of India, and the two inactivated vaccines BBIBP-CorV by Sinopharm and CoronaVac by Sinovac [27]. Several other vaccines against SARS-CoV-2 are licensed in various countries, clinical development of additional vaccine candidates is ongoing and many more are in preclinical stages [28].

The number of publications and amount of data on vaccination against SARS-CoV-2 is immense and grows on a daily basis. A complete overview of SARS-CoV-2 vaccine development is beyond the scope of this article but can be found elsewhere [29]. This review does not claim completeness, but rather aims to highlight aspects specifically relevant to the older population and reflects the state of knowledge in October 2021. An overview of COVID-19 vaccines in children and adolescents has recently been published [30]. 

## 2. Vaccines Licensed in Europe and the US

Within two weeks of reports of a cluster of pneumonia cases with distinct clinical presentation caused by an unknown pathogen in Wuhan, China (31 December 2019) the pathogen was identified as a novel coronavirus, named nCOV2019 at the time (renamed SARS-CoV-2 in February 2020), and the genetic sequence of the virus was made public. Vaccine development was initiated immediately by many companies and organizations worldwide. After tremendous efforts in development and clinical testing, the first vaccines received emergency use authorizations in the US, and conditional marketing authorizations in Europe approximately one year later in late 2020/early 2021. Mass vaccination campaigns started quickly in many countries, but due to limited vaccine supply during the first months, prioritization of who was to be vaccinated first was necessary. As they are at high risk for severe disease, older adults were among the first to receive the vaccines.

### 2.1. Vaccine Types and Status of Regulatory Approval

The first vaccines to be used in the US and Europe were mRNA and viral vector vaccines (Table 1). All four vaccines described in Section 2 deliver genetic information for the SARS-CoV-2 spike protein, which is then translated in the cells of the vaccinee and elicits specific immune responses.

BNT162b2 and mRNA-1273 are mRNA vaccines, which comprise modified mRNAs encoding for the full-length SARS-CoV-2 spike glycoprotein (S) encapsulated in lipid nanoparticles (LNP). Both vaccines are administered intramuscularly in a two-dose regimen. The pivotal trials for licensure were performed with an interval of 21 days (BNT162b2) or 28 days (mRNA-1273) between the two doses. Both vaccines first received emergency use authorizations in the US and conditional marketing authorizations in Europe for use in persons older than 16 (BNT162b2) or 18 years (mRNA-1273). BNT162b2 received full approval in the US for individuals aged ≥16 years at a later time point. Emergency use authorization for this vaccine was extended gradually to adolescents and children and is now granted for children over 5 years. Emergency use authorization for both mRNA vaccines was also issued for the administration of a third dose (short interval) in immunocompromised persons, and for a third dose in persons 65 and older, as well as in younger adults at high risk for severe COVID-19 or with high exposure risk, at least 6 months after the second dose. The conditional marketing authorizations in Europe currently include the use in children older than 12 years for both vaccines, as well as third doses for adults (≥18 years) 6 months after the second dose, and at shorter intervals for immunocompromised persons.

AZD1222 is a viral vector vaccine, which uses a replication-deficient (E1- and E3-deleted) chimpanzee adenovirus (ChAdOx1), which encodes the SARS-CoV-2 spike protein combined with a tissue plasminogen activator (tPA) leader sequence. The vaccine is administered intramuscularly in a two-dose regimen with a time interval of 4–12 weeks between the two doses. AZD1222 received conditional marketing authorization in Europe for adults (≥18 years) but is not authorized in the US. 

COVID-19 vaccine Janssen also uses an adenoviral vector to deliver the genetic information of the SARS-CoV-2 spike protein. A replication-deficient human adenovirus 26 (Ad26, E1-deleted) is used as the vector and encodes the SARS-CoV-2 spike protein. It is also administered intramuscularly, but in contrast to the other vaccines described in this paragraph, Ad26.COV2-S is given as a single dose. Emergency use authorization in the US and conditional marketing authorization in Europe have been granted for use in persons ≥18 years. In October 2021 emergency use authorization in the US was extended to allow administration of a second dose at least 2 months after the first vaccination.

### 2.2. Preclinical Development

Preclinical development in animal models usually includes studies on toxicity and safety, pharmacokinetics, immunogenicity, and protection after infectious challenge. Most studies are performed in young animals, but given the fact that age is a major risk factor for severe disease and, therefore, older adults are an important target group for vaccination, data on immunogenicity and protection in aged animal models would be highly desirable but are scarce. 

It has been reported that immune responses after the second dose of mRNA-1273 are higher than after the first dose in young and aged mice, which is not surprising, and that the IgG-subclass distribution is similar in young and older mice. Two doses of the vaccine protected aged mice from viral replication in the lungs and weight loss after viral challenge. However, no direct comparison with young mice using the same vaccine and viral challenge dose was shown [31]. A detailed study of murine immune responses following immunization with AZD1222 showed that T cell and antibody responses after a single dose of the adenoviral vector vaccine were lower in old compared to young mice, but that a second dose of the vaccine increased the immune response in aged animals [32]. Immunogenicity and challenge studies have also been performed in nonhuman primates (NHP) for all four vaccines, but no aged animals were included in most studies [33]. Only Ad26.COV2-S was tested in aged NHP. It induced similar, potentially slightly lower antibody responses and a similar T cell response profile in aged compared to adult NHP, and aged animals were protected against viral challenge [34].

### 2.3. Immunogenicity

Healthy young adults have a lower risk for severe adverse events and, therefore, early-stage clinical trials are usually performed in this age group. However, as immunosenescence impairs immune responses in older adults, and many vaccines are less immunogenic and efficient in this age group, age restrictions in clinical trials might lessen the informative value of immunogenicity parameters for vaccine candidates to be used in older adults in clinical practice. Clinical development of vaccines against COVID-19 moved quickly to also include older adults. The phase I dose-escalation trial for mRNA-1273 started with participants aged 18–55 years but was extended to also include persons >55 and >70 years [35], and BNT162b2 was tested early in the age groups 18–55 and 65–85 years [36]. In both studies, binding and neutralizing antibodies were similar in the different age groups. In the trial for mRNA-1273, Th_1_-biased CD4^+^ T cell responses were also described to be independent of age. Immunogenicity was also investigated in phase II and III studies, which confirmed these results. More detailed data on immunogenicity became available later, as many studies investigated a plethora of immunological parameters in vaccinees of different age groups. 

In a study investigating humoral and cellular vaccine-induced immune responses, neutralizing antibodies against the original virus were markedly lower in persons older than 80 years compared to younger age groups after the first dose of BNT162b2. This age-associated effect was even more pronounced for virus variants. However, after completion of the two-dose vaccination series, the differences between the age groups were less prominent. Differences between persons younger or older than 80 years were also observed for B cell memory, somatic hypermutation, and T cell responses [37]. 

Immunogenicity of AZD1222 was investigated in persons aged 18–55 y, 56–69 y, and ≥70 y. Antibody levels and neutralizing titers, as well as T cell responses, were similar in the three age groups [38]. The phase I/IIa study for Ad26.COV2-S included two age groups (18–55 y and ≥65 y). Antibody and CD4^+^ T cell responses were slightly lower in the older adults after one dose of vaccine, and antibody levels increased further between day 29 and day 57 after a single dose, suggesting maturation of the B cell response over time. Cytokine-producing CD8^+^ T cells were detectable in 51% of the younger and 36% of the older cohort, respectively indicating a more pronounced decrease of the CD8^+^ T cell responses with age. For the young age group, a two-dose regimen was tested in parallel, leading to higher antibody levels [39]. Nevertheless, Ad26.COV2-S was developed and licensed as a single-dose vaccine. Further immunogenicity trials, including older adults, have been performed for the different vaccines after licensure, and now also include the newly emerging virus variants (see below). These studies also involve specific populations such as older adults in assisted living and long-term care settings. As an example, one study showed that antibody and T cell responses in older adults recruited in an assisted living facility (median age 81 y) were dramatically lower after the first vaccination with BNT162b2 compared to young adults (median age 34 y), and were still significantly lower 4 weeks after the second dose [40]. 

### 2.4. Safety

Age-related aspects of reactogenicity and safety were mainly investigated in later stage trials, which included more older participants. Reactogenicity profiles of the two mRNA vaccines are similar. Pain at the site of injection was observed more frequently than redness and swelling. The most frequent systemic events were fatigue, headache, muscle pain, and chills. [41,42]. Pain and tenderness at the site of injection were also the most frequently reported local signs of reactogenicity for the vector vaccines AZD1222 and Ad26.COV2-S, and the most common systemic reactions were fatigue and headache [38,39,43]. For all vaccines, reactogenicity was higher in younger compared to older adults. This difference was more pronounced for systemic than for local reactions. Interestingly, local and systemic reactogenicity was higher after the first dose of AZD1222 compared to the second dose of this vaccine, but higher after the second than after the first dose of mRNA vaccines. Severe adverse events were only observed in rare cases in all trials and were and mostly not vaccine-related. Very rare severe adverse events were reported after the introduction of the vaccines and the administration to a large number of persons. These reports included thrombosis with thrombocytopenia syndrome (TTS) after vaccination with the adenoviral vector vaccines [44] and allergic reactions, mainly against the mRNA vaccines [45,46]. Rare cases of myocarditis have been observed in adolescents and young adults (<30 y) after vaccination with mRNA vaccines [47]. These rare complications will not be discussed in detail, as they are more relevant for younger adults.

### 2.5. Efficacy in Clinical Trials

The importance of COVID-19 vaccines for older adults was obvious from the beginning, and older adults were included in Phase III clinical trials to determine vaccine efficacy. Overall efficacy of the BioNTech/Pfizer vaccine (two doses, 3-week interval) against symptomatic SARS-CoV-2 infection was 95.0% (95%CI: 90.0–97.9) and did not differ between the age groups 16–55 y, >55 y, and ≥65 y. Forty percent of the participants were older than 55 years in this trial [42]. In the pivotal phase III study for the second mRNA vaccine mRNA-1273, approximately 25% of the participants were older than 65 years. Overall efficacy against symptomatic SARS-CoV-2 infection was 94.1% (95%CI: 89.3–96.8) after two doses administered with a four-week interval. Efficacy was 95.6% (95%CI: 90.6–97.9) for participants younger than 65 years and 86.4% (95% CI: 61.4–95.2) for participants older than 65 years, respectively [41], and a more detailed age-stratification showed an efficacy of 82.4% (95%CI: 46.9–93.9) for persons aged 65–75 y [31]. Both trials also analyzed persons over 75 years of age separately but included only a very limited number of participants in this age group. No cases occurred in the two vaccine arms versus 5 (BNT162b2) and 7 (mRNA-1273) cases in the respective placebo cohorts. Statistical evaluation of efficacy was therefore not possible for this age group [31,42]. 

Several studies with differences in the dosing of the vaccine, time intervals between the two doses, and age of participants were summarized in the report of the clinical trial results for AZD1222, which makes the assessment of the data more complicated. After two full-dose vaccinations, clinical efficacy against symptomatic SARS-CoV-2 infection was 62.1% (95%CI: 41.0–75.7). Only 12% of the participants were older than 55 years and no age-stratified subanalysis was shown [48]. Based on these limited data, some countries recommended this vaccine only for younger adults at the beginning of their vaccination campaigns, but most extended use of the vaccine to older adults at later time points based partially on a press release in March 2021 that announced 79.9% efficacy against symptomatic disease in persons older than 65 years for an ongoing Phase III trial (NCT04516746; [49]). In September 2021, full results of this trial were published and reported an overall efficacy against symptomatic disease of 74.0% (95%CI: 65.3–80.5) and of 83.5% (95% CI: 54.2–94.1) for participants 65 years of age or older [50]. In the pivotal trial for the single-dose of Ad26.COV2-S, in which 33% of the participants were older than 60 years, clinical efficacy against moderate to severe COVID-19 disease was 66.1% (95% CI: 55.0–74.8), without differences between persons younger or older than 60 years [39]. Efficacy data for the mRNA and vector vaccines are summarized in Table 2.

Vaccine efficacy against asymptomatic infection is of particular interest from a public health perspective because viral transmission from vaccinated, asymptomatically infected individuals plays an important role in the overall spread of the virus. Different strategies to detect asymptomatic infections were implemented in the pivotal clinical trials. Weekly self-swabs and PCR testing were performed in a subcohort of the participants in the first AZD1222 studies to detect asymptomatic infection, but efficacy was not statistically significant after receiving two full-dose vaccinations [48]. In a later trial with this vaccine, efficacy against SARS-CoV-2 infection (asymptomatic and symptomatic) was calculated based on the detection of antibodies against the viral N protein and was 64.3% (95% CI: 56.1–71.0) [50]. This assay identifies persons who had been infected, as the N protein is not present in the vaccine. The investigators of the Ad26.COV2-S vaccine determined antibodies against the viral N protein 71 days after vaccination in persons without symptoms and reported a vaccine efficacy against asymptomatic infection of 65.5% (95%CI: 39.9–81.1) [39]. For mRNA-1273, efficacy against asymptomatic infections was determined by RT-PCR tests in the absence of symptoms or detection of N-specific antibodies and was reported to be 63.0% (95%CI: 56.6–68.5) [51]. No age-stratified data were provided.

### 2.6. Early Effectiveness

Vaccine supply was limited at the beginning of national vaccination campaigns, and prioritization of specific risk groups to receive the first available vaccines was implemented. The first groups to be vaccinated included health care workers and older adults. Effectiveness in the “real life” setting was monitored from the beginning in many countries and is still ongoing. In many countries, health care workers are tested for SARS-CoV-2 infection on a regular basis irrespective of their vaccination status. The first data on effectiveness against asymptomatic infections came from studies in these cohorts. High effectiveness of the mRNA vaccines was observed after the first dose and further increased after the second vaccination [52,53], but these studies did not include older adults. The first nationwide data on effectiveness came from Israel, where vaccination with BNT162b2 was rolled out particularly fast, and 90% of the population over the age of 65 years had been vaccinated with two doses by early April 2021. An observational study using national surveillance data from the first 4 months of the nationwide vaccination campaign reported vaccine effectiveness against several outcomes including asymptomatic and symptomatic SARS-CoV-2 infection, COVID-19-related hospitalization, as well as COVID-19-related death, stratified by age (16–44 y; 45–64 y; ≥65 y). Vaccine effectiveness was above 85% for all outcomes and age groups. The lowest value was 85.9% (95%CI: 80.2–89.9) for asymptomatic infection in the oldest age group; most of the other outcomes were above 95%. A more detailed analysis of the oldest age group did not reveal any differences between the age groups ≥65 y, ≥75 y, and ≥85 y [54]. Further studies in other countries, and also with the other vaccines, confirmed that effectiveness mirrors the efficacy outcomes of the clinical trials. A detailed review of vaccine efficacy and effectiveness in the first months of vaccination campaigns can be found elsewhere [55]. It has to be pointed out that the pivotal clinical trials, as well as the first data on effectiveness, only covered the first few months after vaccination, which is, of course, to be expected and justified in a pandemic situation. However, it is unusual compared to data we are familiar with from other vaccines. Waning of immune responses and decreasing protection against at least some outcomes over time was expected, and this aspect is discussed below.

### 2.7. Immune Responses and Effectiveness over Time and in the Presence of Virus Variants

Public interest regarding the duration of protection and, as a consequence, the need and optimal timing for additional vaccine doses, is high. A defined threshold for a correlate of protection would be extremely valuable to monitor protection over time. In addition, such a correlate of protection could facilitate and simplify the development of more vaccines, which are needed for global protection, particularly in low-income and middle-income countries, and of “modified” vaccines that incorporate sequence variants. Neutralizing antibodies have been suggested as correlates of protection by evaluating the relationship between antibody levels and efficacy of several vaccines [56,57]. Circulating antibodies are clearly relevant to prevent infection, but memory B cells, which restart antibody production after contact with the pathogen, and T cells also provide protection against disease. The exact contribution of each immune parameter to protection against different outcomes is still unclear. 

First reports showed that antibodies and T cell responses persist for several months after mRNA vaccination [58,59]. A lower dose of the Moderna mRNA vaccine (25 µg instead of 100 µg) induced slightly lower antibody responses in old compared to younger adults, but no differences in T cell responses were detected between the age groups. Six months after vaccination, immune responses were similar to that of recovered COVID-19 patients [58]. However, most studies describe a slight decline of immune responses within the first 4–6 months after vaccination, and it seems reasonable to assume that this will continue over time. In addition, we have to keep in mind that immune responses and their decline are distinct for different risk groups, e.g., for older adults. It has recently been reported that antibody levels, neutralizing antibodies, and T cell responses 6 months after vaccination with BNT162b2 were substantially lower in older adults enrolled at an assisted living facility compared to young healthcare workers. Only 61% of the older cohort had detectable neutralizing antibodies against the Delta variant 6 months after vaccination compared to 95% for the young participants [60]. Based on neutralizing antibodies as the best available correlate of protection, modeling of the decay of neutralizing titers over the first 250 days post vaccination predicts that a significant loss in protection against viral infection will occur, but that protection against severe disease will be preserved [56]. This finding is in concordance with recent observations regarding clinical effectiveness. 

As mentioned above, the first results of the pivotal Phase III trials only reported efficacy for a very short follow-up of 2–3 months (Table 2), which is unprecedented. Efficacy trials for other vaccines usually collect data for several years in order to reach meaningful numbers of cases. For both mRNA vaccines, final analyses were published at the end of the blinded phase, which included a follow-up of 6 or 5 months for BNT162b2 or mRNA-1273, respectively. Overall vaccine efficacy of BNT162b2 against symptomatic disease was 91.3 % (95%CI: 89.0–93.2) and 94.5% (95% CI:88.3–97.8) for participants older than 65 years [61]. For mRNA-1273 overall efficacy was reported to be 93.2% (95%CI: 91.0–94.8) and 89.7 (95%CI: 79.6–94.9) for participants aged 65–75 years [51], confirming earlier data. Unfortunately, this question cannot be studied for longer periods of time in the context of the placebo-controlled clinical trials, as participants who originally received placebo were offered vaccination in the meantime. A plethora of studies have recently reported, or are still investigating, clinical effectiveness of the different vaccines over time. 

However, analysis of effectiveness over time is much less straightforward than one might think. Various confounding factors need to be taken into account in observational studies. Among the first persons receiving the vaccines were the oldest age groups, and particularly those living in long-term care facilities. The epidemiological situation and, thereby, exposure risks change repeatedly, each vaccine has to be investigated separately, in many countries different age groups receive different vaccines, or, at a given time, a specific vaccine is preferentially administered. In addition, the situation gets even more complex due to new circulating virus variants.

RNA viruses are prone to errors during their genome replication leading to a large number of mutations. If such mutations provide a selection advantage, e.g., due to more rapid spread or partial immune escape, virus variants emerge, which can replace the “older” variants locally or globally. All currently used COVID-19 vaccines are based on the original virus reference sequence (Wuhan-Hu-1; GenBank accession: NC_045512.2), but during the course of the pandemic several virus variants emerged, which replaced the “older” variants locally or globally. Sequence variation occurs throughout the viral genome, but changes in the amino acid sequence of the spike protein are of particular relevance for vaccine effectiveness. The World Health Organization (WHO) has been monitoring and assessing changes in the SARS-CoV-2 genome since the beginning of the pandemic, and during late 2020 established its classification of Variants of Interest (VOI) and Variants of Concern (VOC) to prioritize global monitoring and research. Currently, WHO lists four VOC (Alpha, Beta, Gamma, Delta), all of which were first detected between May and November 2020. The nomenclature changed over time from first reports designating the variants based on their geographical location, to an alpha-numeric designation (Pango lineage), and finally to the Greek letter nomenclature (Table 3). Two variants are currently listed as VOI (Lambda, Mu) and six variants were listed as VOI in the past, but are no longer considered relevant (Kappa, Iota, Eta, Epsilon, Zeta, Theta) [62]. The temporal and spatial distribution of the variants is relevant when discussing efficacy and effectiveness data. The Alpha variant replaced the “parental” virus in many countries in the first months of 2021, and by April/May 2021 more than 90% of the sequences analyzed in Europe and 66% in the US were Alpha. In contrast, Alpha was never dominant in some countries, such as in India, and was rarely found in South Africa and Brazil, where Beta or Gamma, respectively, were circulating almost exclusively at that time. Only a few months later in summer 2021, the Delta variant had replaced all other variants almost completely [63]. A detailed summary of the biological and clinical relevance of SARS-CoV-2 variants has recently been published [64]. 

The antibody responses elicited by the vaccines encoding for the “original” spike protein do not equally bind and neutralize the variant spike proteins. Upon emergence of the different variants, sera from vaccinees have been tested for neutralization of variant SARS-CoV-2 viruses or in different pseudovirus neutralization assays using the variant spike proteins in many studies. It has been shown that antibodies elicited by vaccination with BNT162b2 effectively neutralize the Alpha variant spike, but show a five-fold decrease in the neutralization of the Beta variant [65]. Similar results were reported for the mRNA-1273 vaccine. Neutralization of the Alpha variant was not reduced, whereas neutralization of several other variants was decreased (e.g., 8-fold for Beta, 2-fold for Delta) [66]. Another study reported reduced virus neutralization IC_50_ values for the Alpha, Beta, and Delta variants after vaccination with either BNT162b2 or AZD1222, and an approximately two-fold reduction in neutralizing activity for the antibodies elicited by the adenoviral vaccine compared to the mRNA vaccine [67]. Several of these studies also investigated neutralizing antibodies after the first dose of the two-dose vaccines, and in summary showed that the decrease in neutralization of virus variants is more pronounced after the first dose, highlighting the importance of the second dose for optimal protection. A more comprehensive overview of antibody reactivity towards viral variants can be found elsewhere [68]. Antibodies elicited by a single dose of Ad26.COV2-S showed 5-fold and 3-fold lower neutralizing activity against the Beta and Gamma variant, respectively [69]. No data are available regarding the impact of age on neutralizing activity against virus variants.

Efficacy and effectiveness against the “early” variants Alpha, Beta, and Gamma have been investigated in numerous studies [61,70,71], but a comprehensive summary of all data on the interplay between the different vaccines and the different VOC is beyond the scope of this manuscript. In the following, examples of vaccine efficacy and effectiveness against the now predominant Delta variant are highlighted, and more complete reviews of this topic can be found elsewhere [72,73].

A large retrospective cohort study in the US with almost 3.5 million individuals (median age 45 years) investigated the effectiveness of BNT162b2 over time (up to 6 months after vaccination) and for Delta versus other variants. This was possible due to the timing of the study, which covered December 2020 to August 2021, and thereby the period when Delta gradually replaced the other variants. Effectiveness against SARS-CoV-2 infection (independent of symptoms and for all virus variants) was 88% (95% CI: 86–89) in the first month after two-dose vaccination and declined to 47% (95% CI:43–51) after 5 months for the whole cohort, and from 80% (95% CI: 73–58; first month) to 43% (95%CI: 30–54) for participants older than 65 years. However, efficacy against hospitalization did not show significant waning, and was still 88% (95% CI: 82–92) for the total cohort and 83% (95% CI: 82–92) for the older age group after 5 months. Effectiveness against infection with the Delta variant was lower compared to other variants, (75% (95% CI: 71–78) vs. 91% (95% CI: 88–92) for the total observation period), but the difference in the rate of decline in vaccine effectiveness between Delta and the other variants was not significant. Effectiveness against COVID-19-related hospitalization was similar for Delta (93% (95%CI: 84–96)) and other variants (95% (95%:CI: 90–98)) [74]. Another observational study investigated the vaccine effectiveness of BNT162b2 and AZD1222 (two doses) against symptomatic infection with the Alpha or the Delta variant in the UK. Vaccine effectiveness for the Alpha variant was 93.7% (95%CI: 91.6–95.3) with BNT162b2 and 74.5% (95% CI: 68.4–79.4) with AZD1222, respectively. For the Delta variant, the effectiveness was reduced to 88.0% (95%CI: 85.3–90.1) or 67.0% (95%CI: 61.3–71.8), respectively. This study also confirmed previous reports on immunogenicity, as a single dose of either vaccine provided only limited protection, particularly against Delta [75].

In summary, vaccine-induced immune responses decline over time to a certain extent and recognize some virus variants less than the original virus. Both observations are hardly surprising. Vaccine effectiveness against infection, particularly against asymptomatic infection, decreases over time and is also lower against virus variants. Nevertheless, effectiveness against severe disease or hospitalization is much more robust both over time and in the presence of virus variants. The decline of effectiveness against some outcome parameters that we currently observe probably is a combination of waning immune responses with increasing time since vaccination and the global dominance of the Delta variant. The exact extent of these effects is influenced by the type of vaccine as well as by the immune response of specific cohorts, e.g., older adults. Therefore, data need to be collected and analyzed specifically for these cohorts, and vaccination strategies need to be tailored accordingly.

### 2.8. Vaccination Strategies and Additional Doses

Optimal vaccination schedules are crucial to achieve the highest possible protection. As mentioned above, older adults were among the first to receive vaccination in most countries. However, from the very beginning of the national vaccination campaigns, different countries followed distinct strategies regarding, for example, choice of vaccine for different age groups and time intervals between the two vaccine doses. As an example, some countries in Europe started vaccination of older adults using only mRNA vaccines, as less data for older adults were available at that time regarding AZD1222. Upon reports of very rare severe thrombotic side effects after administration of this vaccine in young adults, several European countries then only used the vaccine for the older population. Decisions regarding the interval between the two vaccine doses were also heterogeneous. At the beginning of vaccination campaigns, when vaccine availability was still very limited, national recommendations tried to find a fair balance between two potential strategies. Option one was the administration of two doses with short intervals, which would lead to optimal protection of the vaccinated individuals quickly. Option two was the administration of the first dose to as many persons as possible and delaying the second dose. This strategy would lead to partial protection of more individuals after the first dose, but, of course, would need more time to complete the vaccination regimen. In addition, there were first hints early after the introduction of the vaccines that longer intervals between the two doses might lead to improved antibody responses and clinical efficacy. The Phase III trials of AZD1222, for instance, included subcohorts for whom the interval between the two doses was longer than originally planned due to changes in the study protocol, and the data suggested increased efficacy with the longer intervals. However, the study was not really powered for this type of subanalysis in the first interim analysis [48]. As more data became available from these trials, clear differences were reported between efficacy against symptomatic COVID-19 after two doses of AZD1222 either less than 6 weeks (55.1% (95%CI: 33.0–69.9) or more than 12 weeks (81.3% (95%CI: 60.3–91.2) apart [76]. It has been reported that an extended time interval between two doses of BNT162b2 (11–12 weeks instead of 3 weeks) increased peak antibody responses of persons older than 80 years approx. 3.5-fold, but at the same time led to decreased cellular responses [77]. At present, vaccine supply is not limited in high-income countries, but national recommendations on vaccine intervals are still quite heterogeneous. It was also suggested early that heterologous prime-boost schedules might be superior to two doses of the same vaccine. First studies have recently been published demonstrating similar or improved immune responses of heterologous vaccination with one dose of AZD1222 followed by one dose of BNT162b2 [78,79,80], but these studies are too small to evaluate the clinical efficacy of the various schedules.

In July 2021, reports from Israel announced only 64% vaccine effectiveness against infection (regardless of symptoms) in the previous weeks (compared to>90% in the early studies; see Section 2.6), while effectiveness against serious illness and hospitalization was still 93% [81]. These reports marked the start of a discussion regarding additional doses. Israel quickly decided to provide persons older than 60 years, who received their second dose of BNT162b2 at least 5 months earlier with a third vaccine dose and extended this recommendation soon thereafter to younger age groups. Reports of waning immune responses and declining effectiveness have accumulated over the last several months (see Section 2.7) and many high-income countries have now implemented strategies to deliver an additional dose of vaccine. The details of exact timing and target groups for the additional doses differ between countries. In general, the recommendations agree to administer the additional dose to risk groups including older adults approximately 6–9 months and (if recommended) to the general population approximately 9–12 months after the second dose (or the single dose of Ad26.COV2-S). Several countries in Europe decided to only, or preferentially, use mRNA vaccines for the additional dose and to vaccinate everybody who initially received an adenoviral vaccine with an additional dose of mRNA vaccine 6 to 9 months later. Some countries now recommend administering a second dose of preferentially mRNA vaccine as soon as possible (>28 days) to individuals who received a single dose of Ad26.COV2-S [82]. In the US, a third dose is recommended for persons with risk factors including adults older than 65 years 6 months after the second dose of an mRNA vaccine, and for all adults 2 months after a single dose of Ad26.COV2-S [83].

It has been shown that the third dose of BNT162b2, mRNA-1273, or AZD1222 induces a robust increase in antibody levels [84,85,86,87]. It has to be mentioned that the third dose of the Moderna vaccine comprises a reduced amount of mRNA (50µg instead of 100µg). First effectiveness data from Israel report an 11.3-fold (95% CI: 10.4–12.3) reduced risk for infection and a 19.5-fold (95% CI: 12.9–29.5) reduction of severe illness for persons over the age of 60 after the third dose of BNT162b2 compared to individuals who had not yet received the third dose [88]. In a second study in Israel, which included younger adults (median age 52 years) effectiveness of the third dose compared with two doses only was described to be 93% (95% CI: 88–97) against hospital admission, and was similar for the age groups 40–69 years and ≥70 years [89].

However, these efforts have to be viewed in light of still extremely poor vaccine supply in many low-income countries. The WHO has cautioned against broad delivery of third doses and advocates strongly for worldwide primary vaccination.

## 3. Conclusions and Outlook

The unprecedentedly fast development of efficient vaccines against SARS-CoV-2 has prevented a large number of deaths and severe cases of disease over the last year, particularly in older adults, and has helped to curtail viral spread in many countries. But we are still far from ending the pandemic.

Vaccine efficacy and effectiveness was extremely high in the first months after vaccination, not only in young but also in older adults. However, despite lasting effectiveness against severe disease and death, vaccine effectiveness against asymptomatic or mild infection declined over time, which has led to uncertainties in the public perception. The public and many stakeholders and governments were under the impression that the pandemic and the need for non-pharmaceutical interventions would end immediately as soon as a certain percentage of a population had been vaccinated. As we have learned over the last months, that is not entirely true. Even though vaccinated persons are still less likely to transmit the disease if they are themselves infected, transmission is possible from vaccinated persons. In addition, a substantial portion of individuals is still unvaccinated in high-income countries. Vaccination for children is gradually being implemented and will reduce this percentage, but in many countries a certain portion of the population is unwilling to get vaccinated. Vaccination rates are generally higher in the older age groups but vary greatly, for instance, within Europe. The European Centre for Disease Prevention and Control (ECDC) reported a median of 71.6% for full vaccination coverage among adults (>18 y) with a range of 21.7–90.5% for the 30 EU/EAA countries and of 85.4% (range 19.4–100%) for adults older than 80 years for the countries where these data were available (data for week 36 2021) [82]. In addition, reports of “breakthrough” infections despite vaccination were somehow interpreted by the public as proof of a failure of the vaccines and a reason not to get vaccinated at all. Vaccines are never 100% protective and, therefore, infections in vaccinated persons will inevitably occur. The question is, how many, or better how few, compared to unvaccinated populations? We need to improve vaccination coverage, which means we need to convince unvaccinated persons to accept the offer to receive a vaccine. In Europe, the US, and other high-income countries vaccine supply is not the problem anymore. Third doses have been administered to persons for whom the first two doses date back more than 6 months and are particularly relevant for older adults, other risk-groups and persons with close contact to risk-groups, e.g., medical personnel. At the moment, we cannot know how long those third doses will be effective, but one can speculate—and from an immunological viewpoint it seems likely—that a vaccination scheme of two doses with a short interval (weeks) followed by a third dose several months later will provide a longer-term protection. For many vaccines we use 2+1 schedules to induce long-lasting immune responses and maybe—or rather hopefully—this will also be the case for COVID-19 vaccines.

The situation is quite different in low-income and middle-income countries. There, vaccine supply, but also logistics for local distribution, infrastructure and personnel for vaccination, and supply of accessory equipment (e.g., syringes), are limited, and we all have to address these issues together to provide protection for everybody worldwide. The pandemic is a global phenomenon, and its control needs to be a global effort.

## Figures and Tables

**Table 1:** Nomenclature of COVID-19 vaccines used in Europe and the US.

Original Designation	Commercial Name	Alternative Names	Company	Vaccine Type
BNT162b2	Comirnaty		BioNTech/Pfizer	mRNA/ lipid nanoparticles
mRNA-1273	Spikevax	COVID-19 vaccine Moderna	Moderna	mRNA/ lipid nanoparticles
AZD1222	Vaxzevria	COVID-19 vaccine AstraZeneca ChAdOx-1-nCoV-19	U Oxford/ AstraZeneca	viral vector (chimpanzee adenovirus)
Ad26.COV2-S		COVID-19 vaccine Janssen	Janssen/ Johnson&Johnson	viral vector (Ad-26)

**Table 2:** Dose intervals and vaccine efficacy of BNT162b2, mRNA-1273, AZD1222, and Ad26.COV2-S in the pivotal Phase III clinical trials.

	Doses (Interval) ^1^	Age Group	Efficacy % ^2^ (95% CI)	Ref.
**BNT162b2**	2 (3 weeks)	all participants	95.0 (90.0–97.9)	[42]
		16–55 y	95.6 (89.4–98.5)	
		>55 y	93.7 (80.6–98.8)	
		≥65 y	94.7 (66.7–99.9)	
		≥75 y	100.0 (−13.1–100.0)	
**mRNA-1273**	2 (4 weeks)	all participants	94.1 (89.3–96.8)	[31,41]
		18–64 y	95.6 (90.6–97.9)	
		≥65 y	86.4 (61.4–95.2)	
		≥75 y	100.0 (NE-100.0)	
**AZD1222 ^3^**	2 (4–12 weeks)	all participants	62.1 (41.0–75.7)	[48]
**Ad26.COV2-S**	1	all participants	66.9 (59.0–73.4)	[39]
		18–59 y	63.7 (53.9–71.6)	
		≥60 y	76.3 (61.6–89.0)	

^1^ in the phase III trial(s); ^2^ against symptomatic COVID-19, for Ad26.COV2-S against moderate/severe COVID-19; ^3^ data for participants receiving two standard doses.

**Table 3:** SARS-CoV-2 Variants of Concern (VOC) as defined by WHO (as of 22 October 2021) [62].

WHO Label	Pango Lineage ^1^	Earliest Documented Samples	Date of VOC Designation
Alpha	B.1.1.7	UK Sep-2020	Dec-2020
Beta	B.1.351	South Africa May-2020	Dec-2020
Gamma	P.1	Brazil Nov-2020	Jan-2021
Delta	B1.617.2	India Oct-2020	May-2021

^1^ Includes all descendent lineages.

## Data Availability

Not applicable.
